# Quantitative Analysis of Eight Triterpenoids and Two Sesquiterpenoids in Rhizoma Alismatis by Using UPLC-ESI/APCI-MS/MS and Its Application to Optimisation of Best Harvest Time and Crude Processing Temperature

**DOI:** 10.1155/2019/8320171

**Published:** 2019-08-14

**Authors:** Yanni Tai, Fuxian Zou, Qiurong Zhang, Jia Wang, Ronghui Rao, Ruihua Xie, Shuisheng Wu, Kedan Chu, Wen Xu, Xiaoyan Li, Mingqing Huang

**Affiliations:** ^1^College of Pharmacy, Fujian University of Traditional Chinese Medicine, Fuzhou 350122, China; ^2^Centre of Biomedical Research & Development, Fujian University of Traditional Chinese Medicine, Fuzhou 350122, China; ^3^Nanping Institute of Agricultural Sciences of Fujian Province, Jianyang 354200, Fujian, China

## Abstract

Rhizoma Alismatis (RA), widely known as “Ze-Xie” in China, is the tuber of *Alisma orientale* (Sam.) Juzep (Alismaceae), a Chinese herbal medicine that has been used to treat hyperlipidemia, diabetes, hypertension, dysuria, and inflammation. In this study, a sensitive and reliable method based on an ultra-performance liquid chromatography (UPLC) couple with two ionisation modes, including electrospray ionisation (ESI) and atmospheric pressure chemical ionisation (APCI) tandem mass spectrometry (MS), namely, UPLC-ESI/APCI-MS/MS was developed and validated to simultaneously determine 8 triterpenoids (ESI mode) and 2 sesquiterpenoids (APCI mode) in RA. Ten marker compounds were analysed with a Waters' CORTECS UPLC C18 column (200 mm × 2.1 m, 1.6 *μ*m) and gradient elution with water (contained 0.1% formic) and acetonitrile within 7 min. The established method was validated for linearity, intra- and interday precisions, accuracy, recovery, and stability. The calibration curve for 10 marker compounds showed good linear regression (*r* > 0.9971). The limits of detection and quantification for analytes were 0.14–1.67 ng/mL and 0.44–5.65 ng/mL, respectively. The relative standard deviations (RSD, %) and accuracy (RE, %) of intra- and interday precisions were less than 3.83% and 1.21% and 3.22% and 1.46%, repeatability and stability for real samples were less than 2.78% and 3.19%, respectively. All recoveries of the 10 marker compounds ranged from 97.24% to 102.49% with RSDs less than 4.05%. The developed method efficiently determined the 10 marker compounds in RA and was subsequently applied to optimise harvest time and crude processing temperature. The result indicated the 90% wilted phase and 70°C (or lower) may be the best harvest time and the processing temperature of RA.

## 1. Introduction

Rhizoma Alismatis (RA), widely known as “Ze-Xie” in China, is the tuber of *Alisma orientale* (Sam.) Juzep (Alismaceae) [1]. RA is widely cultivated in China, Japan, Korea, India, and Europe and has been used as a folk diuretic and hypolipidemic agent. Modern research in pharmacology and therapeutics found that RA possesses hypolipidemic, anti-inflammatory, hypoglycaemic, antihypertensive, antitumor, diuretic, and antifatty liver efficacy [2–8].

The main active ingredients of RA are classified into triterpenoids and sesquiterpenoids [9, 10]. Triterpenoids, such as alisol B 23-acetate, exhibit hypolipidemic, hypoglycaemic, and diuretic effects [2, 6, 7]. Alisol C 23-acetate promotes glucose uptake [2] and anticalculus effect in vitro [2, 11, 12]. Alisol B can resist calcium oxalate crystallisation [11, 12] and promote urination [7]. Alisol A has anticalcium oxalate stone function, which promotes glucose uptake and anti-inflammatory activity [2, 5, 12]. Triterpenoids are usually unstable and can be converted into the other triterpenoids during processing [13, 14]. Sesquiterpenoids (e.g., alismoxide and alismol) usually have remarkable antiallergic activity [15] and anti-inflammatory effects [16, 17]. The sequiterpenoid alismol also has antihypertensive effect [18].

Many methods, such as high-performance liquid chromatography-ultraviolet detection (HPLC-UV) [19], high-performance liquid chromatography with an evaporative light scattering detector (HPLC-ELSD) [20], ultra-performance liquid chromatography (UPLC) [21], high-performance liquid chromatography/diode-array detector/quadrupole-time-of-flight mass spectrometry (HPLC-DAD-Q-TOF MS) [22], ultra-performance liquid chromatography with quadrupole-time-of-flight mass spectrometry (UHPLC-Q-TOF MS) [23], and ultra-performance liquid chromatography-tandem mass spectrometry (UPLC-MS/MS) [24], have been established for the qualitative and quantitative analyses of RA for triterpenoid. Gas chromatography-mass spectrometry (GC-MS) was used in the qualitative analysis of RA for the volatile oil, containing sesquiterpenoids [25]. Nevertheless, these methods are only used for the qualitative or quantitative analysis of one type terpenoid (triterpenoid or sesquiterpenoid). To the best of our knowledge, simultaneous detection of triterpenoids and sesquiterpenoids in RA by UPLC-MS/MS have not been previously reported, which are limited to understand the distribution of two kinds of active terpenoid, especially in different harvest times or the processing temperature of RA samples.

APCI and ESI are two ionisation methods in the MS source. APCI is used to analyse small molecule compounds with medium polarity and some volatile compounds. ESI is used to analyse polar compounds and biomacromolecules (nonvolatile) [17]. So, liquid chromatography coupled with two ionisation modes, electrospray ionisation (ESI) and atmospheric pressure chemical ionisation (APCI) tandem mass spectrometry, namely, UPLC-ESI/APCI-MS, is suitable for the analysis of both triterpenoids and sesquiterpenoids in RA.

On the other hand, in contrast to western medicine, traditional Chinese medicine (TCM) has the characteristics of complex composition (multiple types of chemical components) and low poisonousness. The appropriate harvesting and processing technologies for TCM are crucial to the formation of high-quality TCM. Processing method is the key to the retention of chemical components [26–28]. A folk saying states, “in the season is medicine, after the season is grass” and “march harvesting is *Artemisia capillaris* Thunb., April harvesting is *Artemisia annua* L., and harvesting in May and June is firewood,” such as *Apocynum venetum* L. leaves [29] and *Artemisia annua* L. [30]. Therefore, determining the appropriate time for drug collection is the key to ensuring the quality of medicinal materials. This step directly affects the efficiency of medicinal materials for disease prevention and treatment. However, although some articles investigated the harvesting or processing of RA [31–35], they focused only on triterpenoids such as alisol B 23-acetate or/and alisol A 24-acetate (few involved sesquiterpenoids). Thus, the content accumulation process of the two categories (sesquiterpenoids and triterpenoids) in the growth process and the processing of RA was not fully characterised.

Thus, both sesquiterpenoids and triterpenoids are considered necessary for the quantitative analysis in RA. This work aims to develop an UPLC-ESI/APCI-MS/MS for simultaneous determination triterpenoids and sesquiterpenoids in RA and subsequently apply it to optimise the harvest time and crude processing temperature of RA.

## 2. Materials and Methods

### 2.1. Standards, Reagents, and Materials

**Figure 1:**
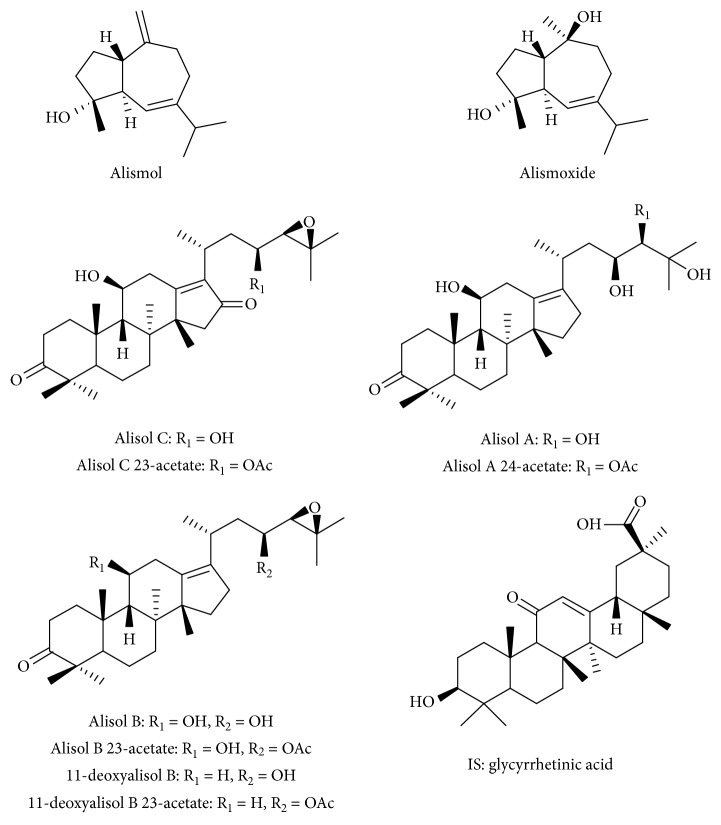
Chemical structures of the 10 investigated compounds and 1 internal standard.

Reference standards of RA alismoxide, alisol C, alisol C 23-acetate, alisol A, alisol A 24-acetate, alisol B, 11-deoxyalisol B, and 11-deoxyalisol B 23-acetate were purchased from Chengdu Mansite Biotechnology Co., Ltd. (Chengdu, China). Alisol B 23-acetate was purchased from National Institutes for Food and Drug Control (Beijing, China). Alismol was purchased from Yunnan Xi Li Biotechnology Co., Ltd. (Yunnan, China). The purity of each standard was higher than 98% by using HPLC-UV and their structures were confirmed by NMR. LC-MS grade acetonitrile (Merck (Darmstadt, Germany)) and formic acid (Sigma-Aldrich, St Louis, MO, USA) were used for chromatographic optimisation. The ultrapure water (18 MΩ/cm) was obtained from Millipore Milli-Q water purification system (Millipore, Bedford, USA). All other reagents were at least of analytical purity and commercially available.  Figure 1 shows the chemical structures of these compounds.

**Figure 2:**
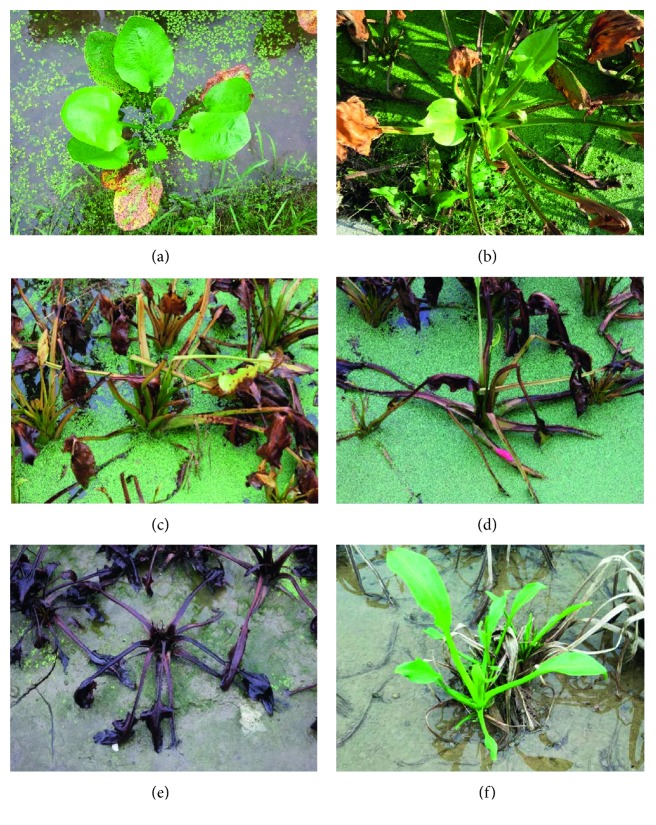
Different wilt stages of RA. (a) Stage I. (b) Stage II. (c) Stage III. (d) Stage IV. (e) Stage V. (f) Stage VI.

A total of 36 batches of harvest season-fresh RA samples were collected at different time points in Nanping, a Good Agricultural Practices (GAP) planting base of RA in Fujian Province by SFDA, China (established in 2001). The sampling method was based on different wilt states. The collection dates and codes were according to GAP conducted by Nanping Institute of Agricultural Sciences of Fujian Province, as follows (Figure 2): Stage I: no wilted, Stage II: 10% wilted, Stage III: 30% wilted, Stage IV: 50% wilted, Stage V: 90% wilted, Stage VI: wilted, sprout regeneration again; Six positions were randomly selected on the same sampling field. At least 0.5 kg of fresh RA was collected per location and wilt states. The husk and fibrous roots of fresh RA were removed and dried under 45°C.

On the other hand, a total of 42 batches of baked samples (processing temperature research) were collected on February 23, 2017. Each of the seven processing groups (freeze dryer, 60°C, 70°C, 80°C, 100°C, 120°C, and 150°C) weighed 30.0 kg (fresh RA) and was randomly divided into six parallels. Then, the fresh RA was placed in a freeze dryer, and others were dried in a heat pump oven at 60°C (60°C–1, 60°C–2, 60°C–3, 60°C–4, 60°C–5, 60°C–6), 70°C (70°C–1, 70°C–2, 70°C–3, 70°C–4, 70°C–5, 70°C–6), 80°C (80°C–1, 80°C–2, 80°C–3, 80°C–4, 80°C–5, 80°C–6), 100°C (100°C–1, 100°C–2, 100°C–3, 100°C–4, 100°C–5, 100°C–6), 120°C (120°C–1, 120°C–2, 120°C–3, 120°C–4, 120°C–5, 120°C–6), and 150°C (150°C–1, 150°C–2, 150°C–3, 150°C–4, 150°C–5, 150°C–6).

All the RA materials were authenticated as tuber of *Alisma orientale* (Sam.) Juzep by medicinal botanist Fan Shi-ming (School of Pharmacy, Fujian University of Traditional Chinese Medicine, Fuzhou 350122), and the voucher specimens were deposited in the School of Pharmacy, Fujian University of Traditional Chinese Medicine. All samples were powdered to a homogeneous size (80 mesh) prior their use.

### 2.2. Preparation of Standard Solution and Samples

Each standard stock solution was prepared separately by dissolving accurate amount of compound in acetonitrile. A series of working solutions of these 10 analytes were freshly prepared by diluting the mixed standard solution with acetonitrile at the ratios of 2, 5, 10, 20, 50, 100, 200, 500, 1000, and 2000 ng/mL. An internal standard stock solution was also prepared in a concentration of 400 ng/mL for glycyrrhetinic acid. All solutions were stored at 4°C before analysis.

The RA samples had a total of 78 batches (including 36 batches of harvest season samples and 42 batches of baked samples). 0.20 g powder was accurately weighted and extracted with 25 mL acetonitrile in an ultrasonic bath (50 kHz, 300 W) for 30 min. Additional acetonitrile was added to make up the lost weight. The extracted solution was centrifuged at 12 000 rpm for 10 min. The supernatant was obtained as a sample solution. A total of 500 *μ*L of the internal standard working solution was added to 500 *μ*L of the mixed standard or sample solution; then, the vortex was blended for 1 min and filtered through a 0.22 *μ*m micropore membrane prior to injection. All the samples were stored at 4°C before analysis.

### 2.3. Chromatographic and Mass Spectrometric Conditions

The UPLC-MS/MS analysis was performed with an ACQUITY UHPLC I-Class system (Waters, Milford, MA, USA) coupled with Xevo TQ–S tandem quadrupole mass spectrometer (Waters, Milford, MA, USA). Data acquisition and quantification were conducted with MassLynx version 4.1 data software (Waters, MA, USA). Chromatographic separation was carried out at 45°C on Waters CORTECS C18 column (2.1 mm × 100 mm, 1.6 *μ*m). 0.10% of formic acid in water was set as the mobile phase A and acetonitrile was set as the mobile phase B. A gradient elution was used as follows: 46%–46% B at 0–0.5 min, 46%–65% B at 0.5–1 min, 65%–90% B at 1–5 min, 90%–100% B at 5–6 min, 100%–46% B at 6–6.1 min, and 46%–46% B at 6.1–7.0 min. The flow rate was 0.25 mL/min, and the sample volume injected was 2 *μ*L. Mass spectrometer conditions were optimised as follows: desolvent gas temperature, 180°C; capillary voltage, 3.5 kV; source temperature, 150°C; desolvent gas flow, 800 L/h; and cone gas flow, 150 L/h. Dwell time was set at 20 ms.

### 2.4. Validation of Quantitative Method

#### 2.4.1. Linearity, LOQs, and LODs

For the calibration curves, at least ten concentrations of calibration standard solution were made and analysed in triplicate. Then, the calibration curve of each analyte was constructed from the peak area ratios of each standard to IS against the concentration of each analyte. The standard solution with the lowest concentration was further diluted to a certain concentration to evaluate the LODs (S/N ratio of 3) and LOQs (S/N ratio of 10), respectively.

#### 2.4.2. Precision, Repeatability, Stability, and Accuracy

The analysis of intra- and interday precisions was carried out by six repetitive injections of a mixed standard solution in the same day and three consecutive days, respectively. Both assays were determined by performing three different concentration levels and LOQs of the standards.

Six RA samples (Stage IV–6) were prepared independently to check the repeatability. To investigate the stability, Stage IV–6 sample solution was analysed within 24 h (0, 2, 4, 8, 12, and 24 h) at room temperature. The recovery was used to evaluate the accuracy of the method and determine by adding the standard solutions with three different concentration levels (low, medium, and high) to the known amounts of RA sample. The percentage recoveries were calculated according to the following equation: (detected amount − original amount) × 100%/spiked amount. The RSD was used to evaluate the results.

## 3. Results and Discussion

### 3.1. Optimisation of Sample Preparation

Different methods were compared to achieve extraction efficiency. The following methods were tested: extraction methods (e.g., ultrasound, reflux, soxhlet, and warm immersion), extraction solvents (e.g., 40%, 60%, 80%, and 100% acetonitrile), extraction time (e.g., 15, 30, 45, and 60 min), and sample-to-solvent ratio (e.g., 1 : 50, 1 : 100, 1 : 125, and 1 : 150). The optimal sample preparation was the extraction of 0.2 g sample with 25 mL of 100% acetonitrile in an ultrasonic water bath for 30 min (Supplementary Materials, Figure S1).

### 3.2. Optimisation of UPLC Conditions

Factors (column, mobile phase, and column temperature) that affected the separation of multicomponent sample were optimised to achieve the simultaneous separation of triterpenoids and sesquiterpenoids. After the comparison of ACQUITY UPLC BEH C18 column (2.1 mm × 100 mm, 1.7 *μ*m) with Waters' HSS T3 C18 column (2.1 mm × 100 mm, 1.8 *μ*m) and Waters' CORTECS C18 column (2.1 mm × 100 mm, 1.6 *μ*m), Waters CORTECS C18 (2.1 mm × 100 mm, 1.6 *μ*m) column was selected for this study because it obtained the best separation efficiency and the most symmetrical chromatographic peaks for the target compounds. In addition, various mobile phases and column temperatures were investigated. Water (contained 0.1% formic) and acetonitrile were selected as the mobile phases because they provided sharp chromatographic peaks and stable baselines.  Table S1 (Supplementary Materials, Table S1) shows the comparison of our methods and the reported methods for analysis of RA.

### 3.3. Optimisation of MS Conditions

For triterpenoids, the ESI-MS spectra were acquired in the multiple reaction monitoring (MRM) mode with a positive electrospray ion source (ESI+). The MRM product ion, collision energy, cone voltage, ion pairs, and the details of the proposed fragmentation pathway of each compound were systematically optimised (Supplementary Materials, Figures S2–S9).

**Figure 3:**
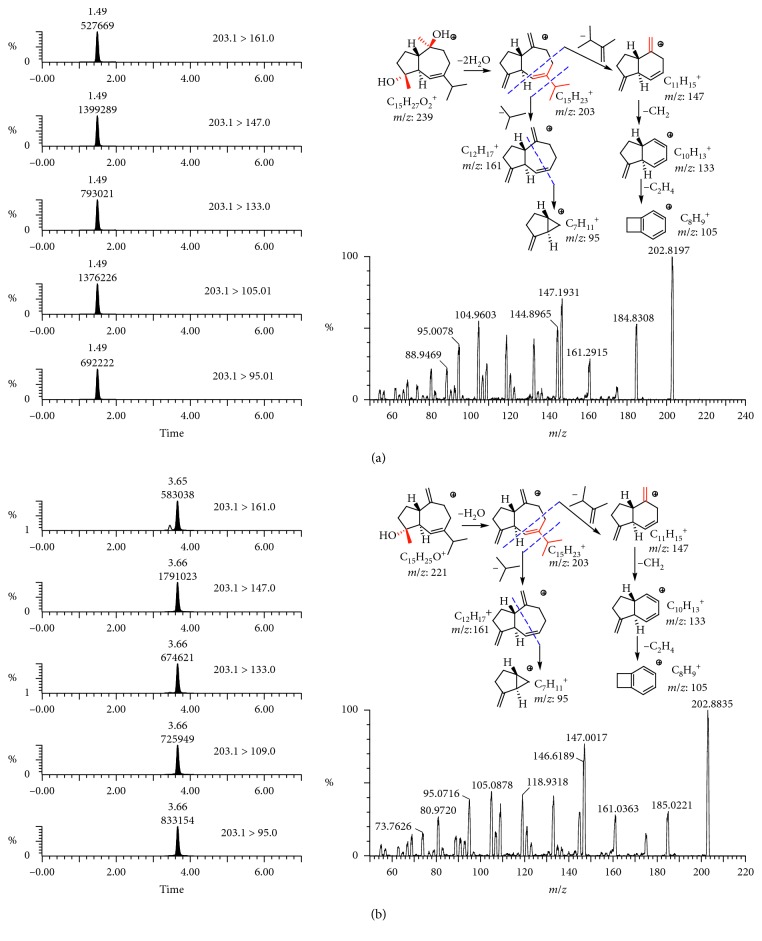
Optimisation of multiple reaction monitoring product ions, APCI-MS/MS spectra and the proposed fragmentation pathway of alismoxide (a) and alismol (b).

The APCI-MS (Figure 3) shows that the MRM product ion, collision energy, cone voltage, and ion pairs were optimised (Figures S10 and S11) to provide the best sensitivity. The positive ion mode was suitable for sesquiterpenoids analyses. The APCI mass spectra gave characteristic quasimolecular ions of alismoxide ([M + H–2H_2_O]^+^ ion at *m*/*z* 203) and alismol ([M + H–H_2_O]^+^ ion at *m*/*z* 203). The ion at *m*/*z* 147 was generated by eliminating C_4_H_8_ (56 Da). Mass spectrometer conditions were optimised as follows: desolvent gas temperature, 180°C; capillary voltage, 3.5 kV; source temperature, 150°C; desolvent gas flow, 800 L/h; and cone gas flow, 150 L/h. Dwell time was set at 20 ms.

**Figure 4:**
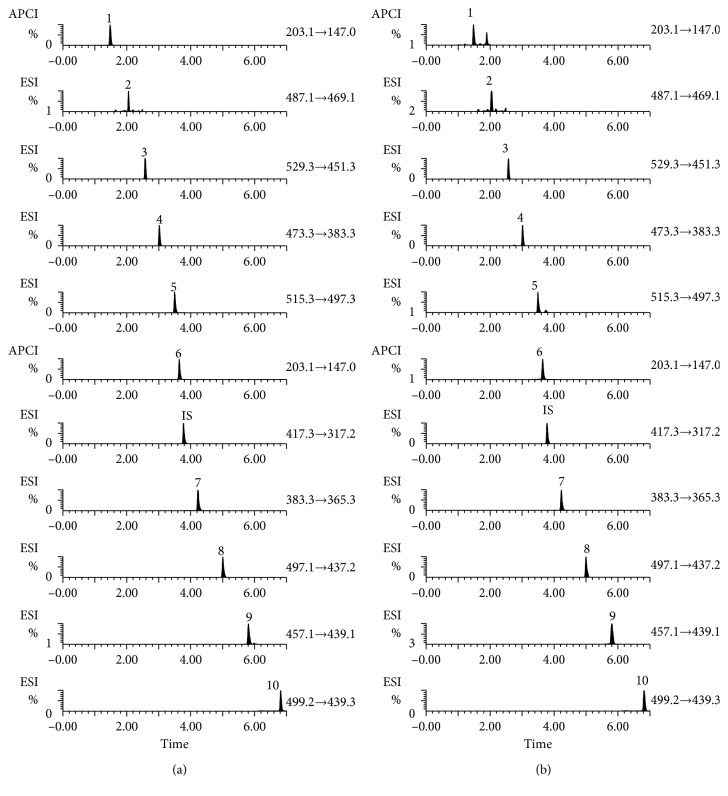
UPLC-ESI/APCI-MS/MS chromatogram of 10 target standards and 1 internal standard of standard solution (a) and RA sample (b). AX: alismoxide; C: alisol C; 23C: alisol C 23-acetate; A: alisol A; 24A: alisol A 24-acetate; AL: alismol; B: alisol B; 23B: alisol B 23-acetate; 11-B: 11-deoxyalisol B; 11-23B: 11-deoxyalisol B 23-acetate.

Retention time, related MS data of the 10 investigated compounds, and internal standards in the UPLC-ESI/APCI-MS/MS analysis were summarized and are shown in Table 1.  Figure 4 shows the optimised MRM chromatogram of the 10 markers.

### 3.4. Method Validation

The UPLC-MS/MS method was validated with precision, linearity, lower limit of quantification (LOQs), lower limit of detection (LODs), repeatability, stability, and recovery.

#### 3.4.1. Linearity, LOQs, and LODs

The calibration curves, which were plotted with at least ten concentrations of standard solutions, were constructed from the peak area ratios of each standard to IS against the concentration of each analyte. The LODs (S/N = 3) and LOQs (S/N = 10) for the 10 standard analytes were in the range of 0.14–1.67 ng/mL and 0.44–5.65 ng/mL, indicating that this method is sensitive for the quantitative analysis in this study (Table 2).

#### 3.4.2. Precision

The precision of the developed method was determined on the basis of intra- and interday variations. For the intraday precision test, the standard solutions were analysed six times, and three different concentrations and LOQs were used in a single day. The solutions for the interday precision test were examined, for 3 days. The relative standard deviations (RSD%) and accuracy (RE%) of intra- and interday precisions were less than 3.83%, 1.21%, and 3.22%, 1.46%, respectively (Supplementary Materials, Table S2).

#### 3.4.3. Repeatability and Stability

Six RA samples (Stage IV–6) were extracted and analysed to confirm their repeatability. The RSD values of 10 analytes were within the range of 0.60%–2.02%. Stability sample solution was analysed within 24 h (0, 2, 4, 8, 12, and 24 h) at room temperature to investigate their stability. Repeatability and stability for real samples were less than 2.78% and 3.19% within 24 h, respectively (Supplementary Materials, Table S3).

#### 3.4.4. Accuracy

Recovery was used to evaluate the accuracy of the method and determine by adding standard solutions with three different concentration levels (low, medium, and high) to the known amounts of RA sample (*n* = 3). The percentage recoveries were calculated according to the following equation:

(1) $$\begin{array}{c} \text{recoveries}=\frac{\left(\text{detected amount}-\text{original amount}\right)\times 100\%}{\text{spiked amount}}. \end{array}$$

Table S4 (Supplementary Materials) shows that the recovery rate of 10 standards varied from 97.24% to 102.49% (RSDs ≤ 4.05%), thereby presenting the acceptable recovery and accuracy of this method.

### 3.5. Optimisation of the Best Harvest Time

**Figure 5:**
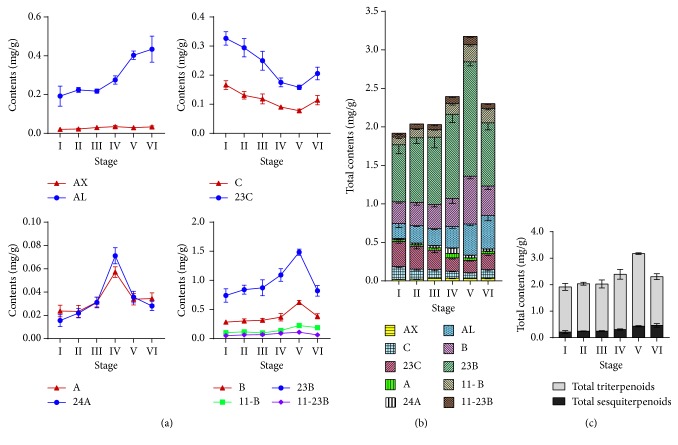
Broken line diagram of the contents of 10 compounds in RA harvesting samples (a). Variation histogram of the total compounds in RA harvesting samples in 6 stages (b). Variation histogram of total triterpenoids and two sesquiterpenoids in the harvesting samples in 6 stages (c). AX: alismoxide, C: alisol C 23C: alisol C 23-acetate, A alisol A 24A: alisol A 24-acetate, AL: alismol, B alisol B 23B: alisol B 23-acetate, 11-B: 11-deoxyalisol B 11-23B: 11-deoxyalisol B 23-acetate.

Table 3 shows the quantification results of these compounds in the 36 batches of RA from different harvest times (different wilt states, Figure 2). Accumulation of 10 analysis compounds is shown in Figure 5. At the beginning of the growth, the contents, such as alisol B, alisol B 23-acetate, 11-deoxyalisol B, and 11-deoxyalisol B 23-acetate metabolites, were low in no wilted state at the initiation stage. Subsequently, the contents rapidly increased to the highest level in the 90% wilted state and then decreased at the stage of wilted, sprout regeneration. For alisol A and alisol A 24-acetate, the content of 50% wilted stage reached the highest level and then decreased. For alisol C and alisol C 23-acetate, the changing trend was almost opposite to alisol Bs, that is, the content of 90% wilted state reached the lowest level. In general, the content of alismoxide and alismol is constantly accumulating during the growth of RA. Combining triterpenoids with sesquiterpenoids, the results showed that sample time of the 90% wilted stage had the highest amount of total compounds (3.180 mg/g). According to the traditional harvesting time of RA [1], it usually occurred in wilted RA, and the result indicated the 90% wilted stage may be the best.

### 3.6. Optimise the Best Processing Temperature

**Figure 6:**
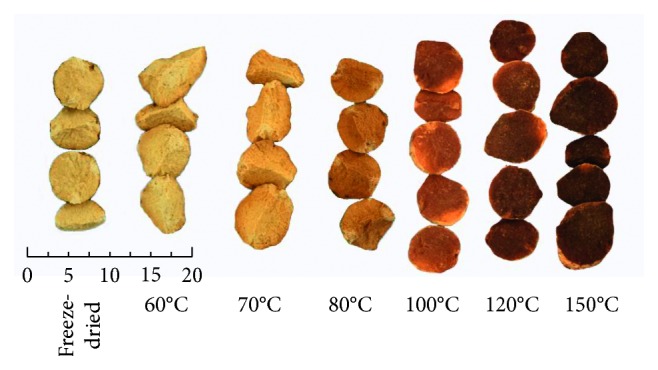
Different processing temperature of RA.

**Figure 7:**
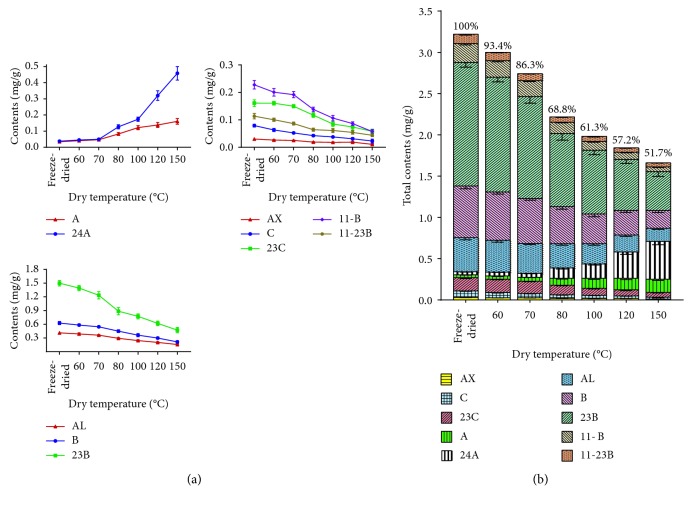
Broken line diagram of the contents of 10 compounds in different RA dry temperature samples (a). Variation histogram of the total compounds in different RA dry temperature samples (b). AX: alismoxide, C: alisol C 23C: alisol C 23-acetate, A alisol A 24A: alisol A 24-acetate, AL: alismol, B alisol B 23B: alisol B 23-acetate, 11-B: 11-deoxyalisol B 11-23B: 11-deoxyalisol B 23-acetate.

Table 4 shows the quantification results of the 42 batch samples (Figure 6) of RA from different dry temperatures. The results (Figure 7) showed that the contents of alismoxide, alisol C, alisol C 23-acetate, alismol, alisol B, alisol B 23-acetate, 11-deoxyalisol B, and 11-deoxyalisol B 23-acetate decreased with the baking temperature. Total contents were decreased with the baking temperature increased, especially when the temperature is above 80°C. Meanwhile, it is obvious that the contents alisol A and alisol A 24-acetate began to generate, and its content is increased with the temperature rise. Analysis of total contents suggested that retention ratio of the total contents is rapidly reduced from 93.4% to 68.8% (lower than 80%) when dried under 80°C, the chemical composition is obviously destroyed by high temperature. When the temperature reaches 150°C, the content of the index component alisol B 23-acetate drops below 0.5 mg/g, the appearance and content of the index component do not meet the requirements of the China Pharmacopoeia. So, we suggested the processing temperature setted at 70°C or lower.

## 4. Conclusions

In summary, a UPLC-ESI/APCI-MS/MS method for simultaneous determination of eight triterpenoids and two sesquiterpenoids in RA has been developed and validated for the first time. MS spectra were acquired in the MRM mode with APCI, and ESI was specifically used for the determination of sesquiterpenoids and triterpenoids, respectively. Then, it is successfully applied to the optimal best harvest time and crude processing temperature to provide basis for the production and processing of RA, the result indicated the 90% wilted phase may be the best harvest time and the processing temperature suggested at 70°C or lower.

## Figures and Tables

**Table 1:** Retention time, related MS data of the 10 investigated compounds, and internal standards in the UPLC-APCI/ESI-MS/MS analysis.

Compounds	*t*_R_ (min)	Fragment ions (*m*/*z*)	Cone voltage (V)	Collision energy (eV)	Ionisation modes
Alismoxide	1.48	203.1 ⟶ 161.0; **203.1 ⟶ 147.0** ^*∗*^; 203.1 ⟶ 133.0; 203.1 ⟶ 105.01; 203.1 ⟶ 95.01	15	15	APCI+
Alisol C	2.05	**487.1 ⟶ 469.1** ^*∗*^; 487.1 ⟶ 451.1; 487.1 ⟶ 415.1; 487.1 ⟶ 379.1; 469.1 ⟶ 451.1; 469.1 ⟶ 415.1; 469.1 ⟶ 379.1	35	15	ESI+
Alisol C 23-acetate	2.57	529.3 ⟶ 511.3; 529.3 ⟶ 469.3; **529.3 ⟶ 451.3** ^*∗*^; 529.3 ⟶ 415.3; 529.3 ⟶ 397.3	30	20	ESI+
Alisol A	3.03	473.3 ⟶ 337.0; 473.3 ⟶ 365.3; **473.3 ⟶ 383.3** ^*∗*^; 473.3 ⟶ 437.3; 473.3 ⟶ 455.3	40	11	ESI+
Alisol A 24-acetate	3.5	**515.3 ⟶ 497.3** ^*∗*^; 515.3 ⟶ 437.3; 515.3 ⟶ 455.3; 515.3 ⟶ 383.3; 515.3 ⟶ 365.3	20	10	ESI+
Alismol	3.64	203.1 ⟶ 161.0; **203.1 ⟶ 147.0** ^*∗*^; 203.1 ⟶ 133.0; 203.1 ⟶ 109.0; 203.1 ⟶ 95.01	10	12	APCI+
Alisol B	4.23	455.1 ⟶ 437.0; 455.1 ⟶ 383.1; 455.1 ⟶ 379.1; **383.3 ⟶ 365.3** ^*∗*^	40	27	ESI+
Alisol B 23-acetate	4.99	497.1 ⟶ 479.1; **497.1 ⟶ 437.2** ^*∗*^; 497.1 ⟶ 419.1; 497.1 ⟶ 383.1; 497.1 ⟶ 365.1	40	8	ESI+
11-Deoxyalisol B	5.81	**457.1 ⟶ 439.1** ^*∗*^; 457.1 ⟶ 385.1; 385.3 ⟶ 367.7	40	10	ESI+
11-Deoxyalisol B 23-acetate	6.68	**499.2 ⟶ 439.3** ^*∗*^; 499.2 ⟶ 385.3; 499.2 ⟶ 421.0; 383.1 ⟶ 367.7	35	12	ESI+
Glycyrrhetnic acid (IS)	3.76	417.3 ⟶ 317.2	35	12	ESI+

^∗^Quantitative ion pair.

**Table 2:** Regression equation, LODs, and LOQs of the 10 investigated compounds.

Compound	Regression equations	Linear range (ng·mL^−1^)	*r*	LODs (ng·ml^−1^)	LOQs (ng·ml^−1^)
Alismoxide	*Y* = 0.0035 *X* + 0.0123	1.021–1021	0.9999	0.16	0.51
Alisol C	*Y* = 0.0430 *X* − 0.0007	4.928–492.8	0.9998	0.82	1.64
Alisol C 23-acetate	*Y* = 0.0105 *X* + 0.0102	5.105–5105	0.9997	0.73	2.55
Alisol A	*Y* = 0.0060 *X* + 0.0087	0.872–872.0	0.9999	0.14	0.44
Alisol A 24-acetate	*Y* = 0.1930 *X* + 0.0005	3.12–3120	0.9990	0.51	1.25
Alismol	*Y* = 0.0051 *X* + 0.0440	3.00–3000	0.9998	0.48	1.51
Alisol B	*Y* = 0.0038 *X* + 0.0216	7.728–7728	0.9985	0.62	3.86
Alisol B 23-acetate	*Y* = 0.0038 *X* + 0.0990	11.3–11300	0.9971	1.67	5.65
11-Deoxyalisol B	*Y* = 0.0009 *X* + 0.0046	3.432–3432	0.9985	0.46	1.72
11-Deoxyalisol B 23-acetate	*Y* = 0.0008 *X* − 0.0004	5.348–5348	0.9997	0.93	1.18

**Table 3:** Contents of the 10 investigated compounds in 36 batches of RA harvesting samples. AX: alismoxide, C alisol C 23C: alisol C 23-acetate, A alisol A 24A: alisol A 24-acetate, AL: alismol, B alisol B 23B: alisol B 23-acetate, 11-B: 11-deoxyalisol B 11-23B: 11-deoxyalisol B 23-acetate.

Sample code	AX	C	23C	A	24A	AL	B	23B	11-B	11-23B
Stage I–1	0.022	0.153	0.288	0.022	0.013	0.172	0.310	0.538	0.094	0.037
Stage I–2	0.017	0.164	0.345	0.025	0.017	0.158	0.290	0.698	0.083	0.070
Stage I–3	0.017	0.195	0.329	0.018	0.012	0.129	0.257	0.845	0.093	0.056
Stage I–4	0.025	0.174	0.351	0.028	0.010	0.212	0.293	0.749	0.110	0.083
Stage I–5	0.015	0.152	0.318	0.021	0.015	0.284	0.266	0.865	0.097	0.090
Stage I–6	0.021	0.173	0.323	0.017	0.015	0.200	0.284	0.752	0.126	0.046

Stage II–1	0.025	0.135	0.246	0.020	0.021	0.216	0.285	0.966	0.116	0.043
Stage II–2	0.018	0.122	0.335	0.020	0.020	0.218	0.246	0.831	0.100	0.057
Stage II–3	0.022	0.119	0.314	0.027	0.020	0.236	0.307	0.829	0.105	0.046
Stage II–4	0.023	0.120	0.276	0.028	0.027	0.239	0.330	0.883	0.116	0.084
Stage II–5	0.028	0.143	0.303	0.022	0.024	0.205	0.291	0.762	0.123	0.115
Stage II–6	0.022	0.147	0.288	0.022	0.024	0.217	0.352	0.767	0.132	0.063

Stage III–1	0.030	0.139	0.238	0.029	0.026	0.226	0.326	0.747	0.104	0.066
Stage III–2	0.032	0.128	0.285	0.035	0.039	0.222	0.303	0.718	0.099	0.059
Stage III–3	0.034	0.111	0.238	0.028	0.031	0.210	0.267	0.802	0.088	0.064
Stage III–4	0.033	0.123	0.216	0.030	0.030	0.219	0.301	1.049	0.104	0.077
Stage III–5	0.034	0.094	0.291	0.029	0.034	0.231	0.326	0.924	0.090	0.108
Stage III–6	0.026	0.123	0.225	0.027	0.034	0.197	0.360	0.998	0.104	0.063

Stage IV–1	0.029	0.090	0.181	0.053	0.077	0.252	0.319	1.034	0.136	0.041
Stage IV–2	0.037	0.087	0.199	0.064	0.081	0.287	0.319	0.999	0.106	0.059
Stage IV–3	0.031	0.088	0.160	0.055	0.070	0.271	0.363	0.993	0.137	0.094
Stage IV–4	0.033	0.084	0.182	0.058	0.075	0.266	0.494	1.253	0.175	0.091
Stage IV–5	0.038	0.097	0.157	0.054	0.062	0.313	0.344	1.201	0.154	0.113
Stage IV–6	0.036	0.092	0.170	0.062	0.068	0.260	0.368	1.078	0.134	0.069

Stage V–1	0.029	0.071	0.150	0.033	0.034	0.376	0.634	1.488	0.216	0.057
Stage V–2	0.030	0.081	0.173	0.031	0.038	0.412	0.610	1.477	0.195	0.062
Stage V–3	0.028	0.073	0.159	0.039	0.037	0.423	0.674	1.399	0.230	0.066
Stage V–4	0.032	0.084	0.156	0.028	0.041	0.379	0.639	1.460	0.252	0.108
Stage V–5	0.028	0.083	0.146	0.032	0.031	0.402	0.565	1.544	0.234	0.121
Stage V–6	0.032	0.080	0.159	0.036	0.033	0.433	0.607	1.538	0.211	0.073

Stage VI–1	0.029	0.131	0.204	0.036	0.028	0.315	0.439	0.742	0.165	0.047
Stage VI–2	0.036	0.124	0.222	0.043	0.032	0.434	0.363	0.819	0.193	0.067
Stage VI–3	0.035	0.102	0.235	0.031	0.024	0.457	0.343	0.828	0.186	0.081
Stage VI–4	0.027	0.134	0.193	0.035	0.034	0.428	0.357	0.701	0.169	0.098
Stage VI–5	0.032	0.105	0.177	0.031	0.031	0.517	0.362	0.882	0.196	0.103
Stage VI–6	0.030	0.100	0.204	0.035	0.030	0.445	0.436	0.949	0.208	0.066

**Table 4:** Contents of the 10 investigated compounds in 42 batches of different RA dry temperature samples. AX: alismoxide, C alisol C 23C: alisol C 23-acetate, A alisol A 24A: alisol A 24-acetate, AL: alismol, B alisol B 23B: alisol B 23-acetate, 11-B: 11-deoxyalisol B 11-23B: 11-deoxyalisol B 23-acetate.

Sample no.	AX	C	23C	A	24A	AL	B	23B	11-B	11-23B
Freeze-dried–1	0.030	0.074	0.146	0.033	0.034	0.393	0.641	1.489	0.230	0.097
Freeze-dried–2	0.030	0.078	0.179	0.031	0.039	0.440	0.626	1.473	0.201	0.120
Freeze-dried–3	0.029	0.085	0.166	0.039	0.040	0.391	0.673	1.420	0.232	0.114
Freeze-dried–4	0.032	0.072	0.163	0.032	0.044	0.382	0.632	1.472	0.242	0.115
Freeze-dried–5	0.028	0.084	0.150	0.033	0.035	0.430	0.567	1.580	0.242	0.125
Freeze-dried–6	0.032	0.080	0.161	0.037	0.034	0.439	0.619	1.549	0.220	0.109

60°C–1	0.026	0.069	0.163	0.042	0.045	0.362	0.588	1.413	0.211	0.093
60°C–2	0.028	0.071	0.169	0.039	0.046	0.417	0.596	1.323	0.200	0.103
60°C–3	0.025	0.059	0.163	0.037	0.046	0.403	0.620	1.477	0.205	0.096
60°C–4	0.026	0.057	0.150	0.031	0.044	0.356	0.577	1.339	0.216	0.101
60°C–5	0.028	0.063	0.156	0.047	0.050	0.396	0.548	1.412	0.195	0.110
60°C–6	0.024	0.060	0.163	0.049	0.041	0.393	0.571	1.381	0.177	0.099

70°C–1	0.025	0.055	0.143	0.045	0.055	0.341	0.516	1.179	0.198	0.089
70°C–2	0.027	0.050	0.154	0.046	0.050	0.367	0.564	1.374	0.206	0.086
70°C–3	0.024	0.055	0.156	0.046	0.042	0.377	0.544	1.248	0.186	0.081
70°C–4	0.025	0.055	0.148	0.044	0.049	0.347	0.570	1.160	0.197	0.089
70°C–5	0.024	0.049	0.143	0.050	0.055	0.372	0.548	1.265	0.174	0.094
70°C–6	0.024	0.050	0.154	0.051	0.044	0.361	0.528	1.184	0.190	0.079

80°C–1	0.020	0.037	0.113	0.085	0.113	0.303	0.411	0.881	0.150	0.064
80°C–2	0.019	0.046	0.128	0.080	0.143	0.268	0.456	0.933	0.139	0.068
80°C–3	0.018	0.044	0.117	0.082	0.140	0.304	0.430	1.017	0.127	0.073
80°C–4	0.016	0.046	0.120	0.099	0.116	0.309	0.491	0.856	0.131	0.062
80°C–5	0.019	0.039	0.104	0.075	0.129	0.296	0.435	0.823	0.141	0.060
80°C–6	0.021	0.043	0.121	0.074	0.120	0.272	0.474	0.806	0.140	0.057

100°C–1	0.019	0.039	0.088	0.131	0.165	0.245	0.376	0.706	0.119	0.063
100°C–2	0.018	0.035	0.083	0.119	0.185	0.272	0.305	0.782	0.107	0.071
100°C–3	0.017	0.038	0.092	0.121	0.190	0.241	0.377	0.830	0.103	0.067
100°C–4	0.015	0.040	0.085	0.138	0.166	0.251	0.362	0.720	0.115	0.059
100°C–5	0.018	0.035	0.069	0.101	0.179	0.233	0.402	0.763	0.092	0.052
100°C–6	0.020	0.039	0.092	0.121	0.157	0.221	0.349	0.836	0.101	0.055

120°C–1	0.019	0.032	0.062	0.116	0.317	0.193	0.286	0.566	0.092	0.060
120°C–2	0.018	0.027	0.078	0.159	0.296	0.234	0.314	0.616	0.088	0.056
120°C–3	0.020	0.035	0.071	0.151	0.302	0.196	0.275	0.570	0.079	0.047
120°C–4	0.021	0.025	0.083	0.139	0.360	0.213	0.292	0.648	0.095	0.063
120°C–5	0.014	0.029	0.088	0.128	0.292	0.189	0.339	0.695	0.081	0.049
120°C–6	0.019	0.037	0.062	0.136	0.353	0.199	0.282	0.622	0.082	0.050

150°C–1	0.012	0.028	0.051	0.169	0.471	0.137	0.227	0.522	0.054	0.048
150°C–2	0.010	0.017	0.070	0.190	0.423	0.172	0.241	0.477	0.067	0.054
150°C–3	0.013	0.012	0.053	0.153	0.473	0.165	0.171	0.505	0.058	0.045
150°C–4	0.010	0.028	0.050	0.161	0.521	0.148	0.225	0.427	0.051	0.048
150°C–5	0.008	0.022	0.064	0.149	0.401	0.168	0.206	0.375	0.065	0.052
150°C–6	0.011	0.029	0.060	0.141	0.460	0.157	0.221	0.523	0.049	0.048

## Data Availability

The data used to support the findings of this study are included within the article and supplementary information file.

## Supplementary Materials

Supplementary Materials Figures S1–S11 and Table S1–S4 show the comprehensive analysis.
