# Supplementary material for: Quantitative Analysis of Eight Triterpenoids and Two Sesquiterpenoids in Rhizoma Alismatis by Using UPLC-ESI/APCI-MS/MS and Its Application to Optimisation of Best Harvest Time and Crude Processing Temperature
Source: J Anal Methods Chem. 2019 Aug 14;2019:8320171. doi: 10.1155/2019/8320171 (PMC6710727; doi:10.1155/2019/8320171)
Supplement: Supplementary Materials — Figures S1–S11 and Table S1–S4 show the comprehensive analysis. [file 8320171.f1.docx]

**Supplementary Material**

**Figure S1** Extraction efficiencies of the 10 investigated compounds by different extractmethods (A); different extraction solvents (B); different extraction time (C); and different times the volume of 100% acetonitrile (D). AX: alismoxide, C: alisol C, 23C: alisol C 23-acetate, A: alisol A, 24A: alisol A 24-acetate, AL: alismol, B: alisol B, 23B: alisol B 23-acetate, 11-B: 11-deoxyalisol B, 11-23B: 11-deoxyalisol B 23-acetate

**Figure S2** Optimisation of multiple reaction monitoring product ions, details of the proposed fragmentation pathway, collision energy and cone voltage of alisol C.

**Figure S3** Optimisation of multiple reaction monitoring product ions, details of the proposed fragmentation pathway, collision energy and cone voltage of alisol C 23-acetate.

**Figure S4** Optimisation of multiple reaction monitoring product ions, details of the proposed fragmentation pathway, collision energy and cone voltage of alisol A.

**Figure S5** Optimisation of multiple reaction monitoring product ions, details of the proposed fragmentation pathway, collision energy and cone voltage of alisol A 24-acetate.

**Figure S6** Optimisation of multiple reaction monitoring product ions, details of the proposed fragmentation pathway, collision energy and cone voltage of alisol B.

**Figure S7** Optimisation of multiple reaction monitoring product ions, details of the proposed fragmentation pathway, collision energy and cone voltage of alisol B 23-acetate.

**Figure S8** Optimisation of multiple reaction monitoring product ions, details of the proposed fragmentation pathway, collision energy and cone voltage of 11-deoxyalisol B.

**Figure S9** Optimisation of multiple reaction monitoring product ions, details of the proposed fragmentation pathway, collision energy and cone voltage of 11-deoxyalisol B 23-acetate.

**Figure S10** Optimisation of multiple reaction monitoring product ions, collision energy and cone voltage of alismoxide using APCI ionisation.

**Figure S11** Optimisation of multiple reaction monitoring product ions, collision energy and cone voltage of alismol using APCI ionisation.

**Table S1** Comparison of our methods and reported methods for quantitative analysis of RA

| References | **Our study** | [1] | [2] | [3] | [4] | [5] | [6] | [7] |
| --- | --- | --- | --- | --- | --- | --- | --- | --- |
| Analysis time (min) | 7 | 8.5 | 39 | 31 | 60 | 50 | 5 | - |
| Analysis marker | 10 | 14 | 8 | 11 | 50 | 7 | 3 | 15 |
| Compounds types | T, S | T | T | T | S | T | T | T, S |
| Analysis types | Ql, Qn | Ql, Qn | Ql, Qn | Ql, Qn | Ql, Qn | Ql | Qn | Ql, |
| LOD (ng/mL) | 0.14-1.67 | 1.01-9.23 | 0.1-4.2 | 0.03-9.09 | - | 5.3-14.1 | - | - |
| solvent consumption (mL) | 1.75 (less than 2.0 mL) | 2.55 | 15.6 | 15.5 | - | 50 | 12 | - |
| Method | UPLC-APCI/ESI-MS/MS | HPLC-DAD-Q-TOF MS and UPLC-QqQ MS | UHPLC-Q-TOF-MS | UHPLC-MS | GC-MS | HPLC-ELSD and HPLC/ESI-MS | LC-MS/MS | 1D and 2D NMR |

“-”: neither qualitative nor quantitative research; “T”: triterpenoids; “S”: sesquiterpenoids; “Ql”: qualitative; “Qn”: quantitative.

**References**

1. Wanli Z, Xiaoqiang H, Xiaoyan L, Fangfang Z, Sainan C, Miao Y, Mingqing H, Wen X, Shuisheng WJM. Qualitative and Quantitative Analysis of Major Triterpenoids in Alismatis Rhizoma by High Performance Liquid Chromatography/Diode-Array Detector/Quadrupole-Time-of-Flight Mass Spectrometry and Ultra-Performance Liquid Chromatography/Triple Quadrupole Mass. molecules 2015; 20: 13958-13981

2. Li HM, Fan M, Xue Y, Peng LY, Wu XD, Liu D, Li RT, Zhao QS. Guaiane-Type Sesquiterpenoids from Alismatis Rhizoma and Their Anti-inflammatory Activity. Chemical & pharmaceutical bulletin 2017; 65: 403-407

3. Gao X, Sun C, Yu Z, Cang J, Tian X, Huo X, Feng L, Liu X, Wang C, Zhang BJAPSB. Correlation analysis between the chemical contents and bioactivity for the quality control of Alismatis Rhizoma. Acta Pharmaceutica Sinica B 2018; 8: 242-251

4. Miyazawa M, Yoshinaga S, Kashima Y, Nakahashi H, Hara N, Nakagawa H, Usami AJJoOS. Chemical Composition and Characteristic Odor Compounds in Essential Oil from Alismatis Rhizoma (Tubers of *Alisma orientale*). Journal of Oleo Science 2016; 65: 91-97

5. LL C, ZF H, XP D, J Q, DN Z, International YBJJoA. Identification and determination of the major triterpenes in Rhizoma Alismatis by HPLC-evaporative light scattering detection and HPLC/electrospray ionization-MS. Journal of AOAC International 2013; 96: 260-264

6. Cheng Z, Ding C, Li Z, Song D, Yuan J, Hao W, Ge QJJoCB. Simultaneous determination of three triterpenes in rat plasma by LC–MS/MS and its application to a pharmacokinetic study of Rhizoma Alismatis extract. Journal of Chromatography B 2016; 1008: 32-37

7. Hong-Guang J, Qinglong J, Kim A, Ryun, Hyemin C, Je Hyun L, Yeong Shik K, Gun LD, Eun-Rhan WJAoPR. A new triterpenoid from *Alisma orientale* and their antibacterial effect. Archives of Pharmacal Research 2012; 35: 1919-1926

**Table S2.** Precision and accuracy of 10 investigated compounds.

| Compounds | Concentration (ng/mL) | Intra-day (%) | | Inter-day (%) | |
| --- | --- | --- | --- | --- | --- |
|  |  | Precision  (RSD) | Accuracy  (RE) | Precision  (RSD) | Accuracy  (RE) |
| alismoxide | 0.51 | 3.06 | 0.15 | 3.22 | 0.12 |
|  | 1.02 | 2.47 | -0.62 | 2.67 | -1.52 |
|  | 20.4 | 3.83 | 0.57 | 1.39 | -0.2 |
|  | 408 | 1.84 | -1.29 | 0.66 | -0.4 |
| alisol C | 1.64 | 2.27 | -0.91 | 2.72 | -1.93 |
|  | 3.28 | 1.92 | -2.13 | 0.78 | -0.76 |
|  | 65.6 | 1.35 | 1.04 | 0.54 | 1.11 |
|  | 1312 | 0.58 | 0.22 | 0.52 | 0.17 |
| alisol C 23-acetate | 2.55 | 2.35 | -0.93 | 2.35 | -0.93 |
|  | 5.1 | 2.26 | -1.11 | 2.75 | -0.69 |
|  | 102 | 1.63 | -1.55 | 1.02 | -0.93 |
|  | 2040 | 0.74 | -0.39 | 0.78 | -0.29 |
| alisol A | 0.44 | 2.25 | -0.62 | 2.15 | 1.28 |
|  | 0.88 | 1.07 | 0.56 | 0.89 | 0.16 |
|  | 17.6 | 2.19 | -0.2 | 2.13 | 0.14 |
|  | 352 | 1.63 | 0.76 | 1.09 | 0.11 |
| alisol A 24-acetate | 1.25 | 3.07 | -0.86 | 2.76 | -0.6 |
|  | 2.5 | 1.9 | -1.07 | 2.31 | -1.34 |
|  | 50 | 2.15 | -0.48 | 2.3 | 1.46 |
|  | 1000 | 1.6 | -0.15 | 1.43 | 0.46 |
| alismol | 1.51 | 1.73 | -2.16 | 2.86 | -1.94 |
|  | 3.02 | 1.98 | -1.55 | 2.89 | -1.38 |
|  | 60.4 | 1.22 | -1.07 | 1.43 | -0.04 |
|  | 1208 | 0.21 | -0.08 | 0.26 | 0.15 |
| alisol B | 3.86 | 1.21 | -0.39 | 1.21 | -0.39 |
|  | 7.72 | 1.1 | -0.56 | 1.46 | -0.07 |
|  | 154.4 | 0.62 | 0.37 | 1.82 | -0.82 |
|  | 3088 | 0.23 | -0.5 | 0.24 | -0.26 |
| alisol B 23-acetate | 5.65 | 1.83 | -1.12 | 3.04 | -0.03 |
|  | 11.3 | 1.99 | -1.92 | 1.66 | -2.49 |
|  | 226 | 1.58 | -2.06 | 1.86 | -0.72 |
|  | 4520 | 1.42 | 0.98 | 0.69 | 0.25 |
| 11-deoxyalisol B | 1.72 | 1.38 | -0.29 | 2.01 | -1.16 |
|  | 3.44 | 2.38 | 1.21 | 1.81 | 0.44 |
|  | 68.8 | 1.55 | -0.83 | 0.7 | -1.09 |
|  | 1376 | 0.86 | -0.08 | 0.77 | -0.28 |
| 11-deoxyalisol B 23-acetate | 1.18 | 3.8 | 1.11 | 2.61 | -0.44 |
|  | 2.36 | 2.59 | -1.27 | 1.89 | -1.7 |
|  | 47.2 | 1.86 | 0.27 | 2.02 | 0.06 |
|  | 944 | 0.6 | 0.71 | 0.52 | 0.64 |

RSD is expressed as measured standard deviation conc. / mean conc. × 100%;

RE is expressed as [measured conc. / nominal conc.)-1] × 100%

**Table S3.** Repeatability and stability for real samples.

| Compounds | Stability  (RSD, %, *n* = 6) | Repeatability  (RSD, %, n = 6) |
| --- | --- | --- |
| alismoxide | 1.2 | 1.24 |
| alisol C | 2.78 | 1.57 |
| alisol C 23-acetate | 1.86 | 3.15 |
| alisol A | 2.07 | 1.69 |
| alisol A 24-acetate | 1.18 | 1.81 |
| alismol | 1.62 | 2.77 |
| alisol B | 0.37 | 2.57 |
| alisol B 23-acetate | 0.56 | 3.19 |
| 11-deoxyalisol B | 1.17 | 2.67 |
| 11-deoxyalisol B 23-acetate | 1.46 | 1.01 |

**Table S4. Recovery data of the proposed method**

| Compounds | Level | Original (μg) | Spiked (μg) | Detected (μg) | Recovery (%) | RSD (%) |
| --- | --- | --- | --- | --- | --- | --- |
| alismoxide | low | 3.790 | 1.78 | 5.643 | 102.15 | 2.12 |
|  |  | 3.743 | 1.81 | 5.538 |  |  |
|  |  | 3.826 | 1.79 | 5.674 |  |  |
|  | medium | 3.729 | 3.63 | 7.178 | 97.24 | 1.78 |
|  |  | 3.823 | 3.59 | 7.322 |  |  |
|  |  | 3.697 | 3.57 | 7.239 |  |  |
|  | high | 3.672 | 5.41 | 9.313 | 100.64 | 3.43 |
|  |  | 3.894 | 5.30 | 8.982 |  |  |
|  |  | 3.711 | 5.36 | 9.159 |  |  |
| alisol C | low | 9.703 | 4.94 | 14.435 | 99.97 | 3.01 |
|  |  | 9.547 | 4.63 | 14.340 |  |  |
|  |  | 9.400 | 4.78 | 14.643 |  |  |
|  | medium | 9.584 | 9.43 | 18.569 | 101.49 | 4.05 |
|  |  | 9.786 | 9.52 | 19.730 |  |  |
|  |  | 9.970 | 9.26 | 19.127 |  |  |
|  | high | 9.795 | 14.53 | 23.884 | 100.27 | 2.71 |
|  |  | 9.464 | 13.87 | 23.462 |  |  |
|  |  | 9.501 | 13.50 | 23.522 |  |  |
| alisol C 23-acetate | low | 17.878 | 8.30 | 26.498 | 102.49 | 2.10 |
|  |  | 17.657 | 8.06 | 25.673 |  |  |
|  |  | 18.047 | 8.58 | 26.983 |  |  |
|  | medium | 17.589 | 17.43 | 34.781 | 100.17 | 2.63 |
|  |  | 18.030 | 16.98 | 34.669 |  |  |
|  |  | 17.437 | 17.19 | 35.292 |  |  |
|  | high | 17.318 | 25.70 | 41.805 | 99.18 | 3.34 |
|  |  | 18.370 | 26.10 | 45.351 |  |  |
|  |  | 17.504 | 25.23 | 42.452 |  |  |
| alisol A | low | 6.546 | 2.96 | 9.405 | 98.60 | 2.97 |
|  |  | 6.465 | 3.24 | 9.591 |  |  |
|  |  | 6.608 | 3.13 | 9.824 |  |  |
|  | medium | 6.440 | 6.25 | 12.447 | 99.35 | 3.36 |
|  |  | 6.602 | 6.38 | 12.854 |  |  |
|  |  | 6.384 | 6.70 | 13.348 |  |  |
|  | high | 6.341 | 9.38 | 16.024 | 101.13 | 3.60 |
|  |  | 6.726 | 9.45 | 16.568 |  |  |
|  |  | 6.409 | 9.37 | 15.404 |  |  |
| alisol A 24-acetate | low | 7.141 | 3.45 | 10.634 | 100.33 | 2.36 |
|  |  | 7.053 | 3.38 | 10.523 |  |  |
|  |  | 7.209 | 3.42 | 10.529 |  |  |
|  | medium | 7.026 | 6.84 | 13.596 | 98.15 | 2.55 |
|  |  | 7.202 | 6.83 | 14.146 |  |  |
|  |  | 6.965 | 6.64 | 13.388 |  |  |
|  | high | 6.918 | 10.30 | 17.732 | 101.34 | 2.61 |
|  |  | 7.338 | 11.13 | 18.487 |  |  |
|  |  | 6.992 | 9.87 | 16.749 |  |  |
| alismol | low | 27.372 | 12.54 | 39.849 | 102.22 | 2.01 |
|  |  | 27.034 | 13.43 | 40.830 |  |  |
|  |  | 27.631 | 12.68 | 40.875 |  |  |
|  | medium | 26.930 | 26.49 | 54.248 | 101.34 | 3.75 |
|  |  | 27.605 | 26.28 | 52.846 |  |  |
|  |  | 26.696 | 25.17 | 53.083 |  |  |
|  | high | 26.515 | 37.73 | 66.125 | 100.68 | 3.29 |
|  |  | 28.125 | 38.50 | 65.439 |  |  |
|  |  | 26.800 | 38.62 | 65.478 |  |  |
| alisol B | low | 38.768 | 18.68 | 56.742 | 100.10 | 3.60 |
|  |  | 38.290 | 18.64 | 56.774 |  |  |
|  |  | 39.136 | 18.15 | 58.170 |  |  |
|  | medium | 38.142 | 37.32 | 76.739 | 100.23 | 2.92 |
|  |  | 39.099 | 36.85 | 74.604 |  |  |
|  |  | 37.811 | 37.10 | 75.237 |  |  |
|  | high | 37.554 | 56.18 | 92.175 | 99.37 | 3.75 |
|  |  | 39.834 | 56.80 | 94.514 |  |  |
|  |  | 37.959 | 49.69 | 89.938 |  |  |
| alisol B 23-acetate | low | 113.585 | 54.70 | 166.349 | 97.28 | 2.09 |
|  |  | 112.184 | 53.93 | 163.584 |  |  |
|  |  | 114.663 | 54.30 | 169.001 |  |  |
|  | medium | 111.753 | 108.53 | 215.238 | 99.02 | 2.87 |
|  |  | 114.555 | 109.53 | 223.484 |  |  |
|  |  | 110.783 | 108.69 | 221.937 |  |  |
|  | high | 110.029 | 164.84 | 282.584 | 99.34 | 4.03 |
|  |  | 116.710 | 163.49 | 272.098 |  |  |
|  |  | 111.214 | 162.94 | 271.385 |  |  |
| 11-deoxyalisol B | low | 14.137 | 6.84 | 20.874 | 102.23 | 2.61 |
|  |  | 13.963 | 6.56 | 20.822 |  |  |
|  |  | 14.271 | 6.64 | 21.153 |  |  |
|  | medium | 13.909 | 14.52 | 28.725 | 100.66 | 3.75 |
|  |  | 14.258 | 13.84 | 27.475 |  |  |
|  |  | 13.789 | 13.60 | 27.993 |  |  |
|  | high | 13.695 | 21.54 | 35.753 | 101.01 | 2.69 |
|  |  | 14.526 | 21.95 | 35.864 |  |  |
|  |  | 13.842 | 19.35 | 33.853 |  |  |
| 11-deoxyalisol B 23-acetate | low | 7.318 | 3.46 | 10.694 | 99.84 | 3.07 |
|  |  | 7.227 | 3.62 | 10.765 |  |  |
|  |  | 7.387 | 3.97 | 11.523 |  |  |
|  | medium | 7.200 | 7.53 | 14.906 | 99.96 | 3.33 |
|  |  | 7.380 | 7.21 | 14.754 |  |  |
|  |  | 7.137 | 6.84 | 13.653 |  |  |
|  | high | 7.088 | 11.62 | 18.836 | 98.04 | 2.28 |
|  |  | 7.519 | 10.52 | 17.745 |  |  |
|  |  | 7.165 | 12.49 | 19.132 |  |  |
